# WYSIWYG: IoT Device Identification Based on WebUI Login Pages

**DOI:** 10.3390/s22134892

**Published:** 2022-06-29

**Authors:** Ruimin Wang, Haitao Li, Jing Jing, Liehui Jiang, Weiyu Dong

**Affiliations:** 1 State Key Laboratory of Mathematical Engineering and Advanced Computing, Zhengzhou 450000, China; wrm2013nian@163.com (R.W.); lihaitao_ht@163.com (H.L.); jingjing_cs@hotmail.com (J.J.); jiangliehui@163.com (L.J.); 2 Key Laboratory of Cyberspace Situation Awareness of Henan Province, Zhengzhou 450000, China

**Keywords:** IoT, WebUI, device identification, ensemble learning model, OCR

## Abstract

With the improvement of intelligence and interconnection, Internet of Things (IoT) devices tend to become more vulnerable and exposed to many threats. Device identification is the foundation of many cybersecurity operations, such as asset management, vulnerability reaction, and situational awareness, which are important for enhancing the security of IoT devices. The more information sources and the more angles of view we have, the more precise identification results we obtain. This study proposes a novel and alternative method for IoT device identification, which introduces commonly available WebUI login pages with distinctive characteristics specific to vendors as the data source and uses an ensemble learning model based on a combination of Convolutional Neural Networks (CNN) and Deep Neural Networks (DNN) for device vendor identification and develops an Optical Character Recognition (OCR) based method for device type and model identification. The experimental results show that the ensemble learning model can achieve 99.1% accuracy and 99.5% F1-Score in the determination of whether a device is from a vendor that appeared in the training dataset, and if the answer is positive, 98% accuracy and 98.3% F1-Score in identifying which vendor it is from. The OCR-based method can identify fine-grained attributes of the device and achieve an accuracy of 99.46% in device model identification, which is higher than the results of the Shodan cyber search engine by a considerable margin of 11.39%.

## 1. Introduction

Currently, the Internet of Things (IoT) devices, such as industrial control and wearable devices, are becoming an important part of cyberspace and play an indispensable role in daily human life. However, due to the limitation of hardware resources, IoT devices usually cannot integrate sophisticated security detection or protection mechanisms and tend to be victims of cyber attacks [1]. Meanwhile, due to the lack of management, compromised IoT devices may lose control or be abused as a foothold for further network attacks for a long time before being noticed [2]. The situation poses a nonnegligible security problem to cyberspace as the scale of IoT grows continuously. Device identification is the basis of almost all kinds of cybersecurity management in that it helps administrators to survey the types of IoT devices in the networks, spot devices with vulnerabilities, and assess the risk level of the network, thus supporting security operations such as asset assessment, vulnerability defense, and situational awareness. Therefore, identifying IoT devices correctly is of great importance for improving the security level of cyberspace.

The device identification process can be divided into two stages, namely, data acquisition and type discrimination. Data acquisition may be conducted using active probing or passive snooping. Active probing sends requesting messages to devices according to a specific protocol and extracts information like banners, HTTP headers, HTML pages, and certificates from the response messages. Passive snooping intercepts and saves inward and outward traffic of devices passing through gateways. Type discrimination can be conducted by employing fingerprint matching or machine learning. Fingerprint matching discriminates devices by searching specific signatures in information obtained during the data acquisition stage, while Machine Learning (ML) tries to learn some models from history traffic data and use it for device type classification.

The outcome of device identification may be represented by a multi-dimensional vector composed of vendor, type, model, etc., which can be used to refer to a sort of device uniquely. For example, “Cisco” or “MikroTik” are sample values for vendor dimension, “printer” or “camera” for type dimension, and “RT-AC1200GU” or “SBR-AC1900P” for model dimension. Information about vendor, type, and model is precious in the scenarios such as vulnerability patching and security alerting.

For the fingerprint matching method, a fingerprint usually is a binding of a regular expression (RE) and a set of device attributes. For device identification, the RE is used to match a specific pattern against the message obtained from the device, and if matching, the attributes associated with the RE will be returned as an ID of the device. The fingerprint matching method has been adopted by some typical tools, such as Nmap [3], Zmap [4], and SinFP [5], and by some arising cyber search engines, such as Shodan [6], Censys [7], and ZoomEye [8]. Feng et al. [9] implemented an acquisitional rule-based engine that uses the association rule algorithm in data mining to automatically generate fingerprints. The advantage of the fingerprint matching method is its high certainty and accuracy. However, the drawbacks of the method are also noteworthy in that the construction of fingerprints requires a lot of manual effort, and with the increasing security awareness of vendors, many devices prefer not to provide useful information for identification in their response message to active probing.

Methods of ML try learning some classification models from physical or traffic features. Physical features may be microscopic deviations in the clock skews [10,11] or Radio Frequency [12,13], while traffic features may be characteristics manifested by timestamp or length of datagrams. Recently, device identification by learning from traffic features has become a hotspot of research. The related supervised ML algorithms [14] include Random Forest (RF) [15,16,17,18], K-Nearest Neighbors (KNN) [19], Support Vector Machine (SVM) [20,21,22], Gradient boosting [23], Naive Bayes [24,25], Decision Trees (DT) [26], NLP [27], and so on. The related unsupervised ML methods include the Gibbs Sampling Dirichlet Multinomial Mixture Model (GSDMM) [28], Density-Based Spatial Clustering of Applications with Noise (DBSCAN) [29], Kmeans [30], and so on. Additionally, there are some multi-stage ML methods for device type identification [31,32,33,34]. These ML algorithms heavily rely on the selection of traffic features, which in turn rely on the prior knowledge of domain experts, so it is not very friendly for inexperienced researchers. Deep learning (DL), an important branch of ML, uses end-to-end learning to automatically learn implicit features from data. For example, Neural Networks (NNs) [35,36] and CNN [37,38,39,40] can automatically learn features from raw traffic data of devices, eliminating manual feature selection. However, a single DL model may not fit all datasets, so ensemble learning models [41] have been proposed and have obtained higher classification accuracy than the individual model [42,43,44,45].

We argue that the existing research on device identification has some blanks or shortcomings. Firstly, the data currently used for device identification is mainly from the banner, HTML source code, etc.; there is no work found yet using device login page screenshots as the data source for device identification. Secondly, most research works try classifying a device into vendors that appear in the labeled training dataset, even if the vendor of the device is outside the dataset. A few studies can determine whether a device is from a vendor in the labeled training dataset or from an outside-world unknown vendor, but the conclusion is made only by referring to an empiric threshold in the SoftMax layer. These works suffer from poor accuracy as a higher threshold may lead to higher false negatives, while a lower threshold to higher false positives. Finally, existing studies heavily rely on the signature text in the traffic to discriminate the device type or model but are likely to fail since such information is rare in traffic.

It is observed that IoT devices usually provide WEB UIs for management, whose login pages have distinct characteristics such as logos or layouts specific to vendors or products. Furthermore, some login pages may display the device type and model directly on it, which can act as the signature for identifying the device at the fine-grained level. Inspired by the above observations, we propose a device identification framework based on ML and text extracted from device login page screenshots. At first, we grab login page screenshots as datasets through WEB UIs of devices and label them automatically with vendor information obtained from SSL certificates. Next, we train a CNN with a labeled dataset to learn implicit features from those screenshots and get a multi-classifier for device vendors and append a DNN to the CNN, particularly to get a bi-classifier for better distinguishing vendors that appear in the labeled dataset (known vendors) from those vendors from the outside world (unknown vendors). Eventually, we leverage the OCR technique to extract text strings from the screenshots and search them in a pre-built corpus of IoT devices for fine-grained device attribute identification.

The contributions to this paper are as follows.

(1) We propose a novel method for IoT device identification, which uses commonly available login pages with distinctive characteristics specific to vendors as the data source and transforms the device identification into an image classification problem and can be used as an alternative measure for device identification.
(2) We present an ensemble learning model for device identification. Given a device, the model can firstly judge it from a vendor that appeared in the training dataset, with an accuracy of 99.1%, and if the answer is positive, then identify which vendor it is from, with an accuracy of 98%.
(3) We develop an OCR-based method for extracting and identifying type/model attributes from login page screenshots, with an accuracy of 99.46% in device model identification, which overcomes the shortcoming of the traditional method in the difficulty of obtaining device model information from protocol responses or network traffic.

The rest of this paper is organized as follows. Section 2 describes the related work, Section 3 details the framework for device identification based on device login pages, and Section 4 analyzes the feasibility of the approach in this study. The experimental results are given in Section 5. Finally, Section 6 concludes the paper.

## 2. Related Works

We give a brief review of prior relevant studies from three aspects: webpage-based device identification, unknown class identification, and granularity of device identification.

### 2.1. Webpage-Based Device Identification

Li et al. [20,21] proposed a framework based on the content characteristics of device web pages for surveillance device recognition. Firstly, the framework uses an HTML/XML parser to extract the content of the web page of the device and convert it into textual data. Then, it uses natural language processing technology to perform word splitting, stemming, and redundant content removal to obtain a word vector. Next, it uses a feature selection algorithm to automatically select features from the word vector. Finally, the supervised machine learning algorithm is used to train bi-classifier to judge whether a device is of the surveillance kind. Like [21], the Web-IoT Detection (WID) Tool [27] is written in Python using Selenium, which not only crawls the index page of the device but also recursively crawls sub-URLs. It then uses the *Beautiful Soup* library to extract data from HTML or XML files to identify information such as the model number on the device page and the firmware version running on the device. The above research has some similarities with our work, but there are the following deficiencies. Firstly, the device classification based on textual features in web pages may lead to a decline in accuracy when the features in web pages exist in other styles such as images; secondly, the granularity of equipment identification is not detailed enough, which can only judge whether the device belongs to surveillance type; Thirdly, a lot of manual annotation work needs to be done on the training data set to meet the needs of supervised learning. The framework proposed in this paper can effectively make up for the deficiencies of the above research. Firstly, we conduct device identification based on the screenshot of the Web UI login page, and whether the features are given in the form of text or image, they can be captured by image classification or OCR technology; secondly, the paper can give the vendor, type, and model of the device simultaneously, which is more practical in device identification application; Thirdly, the paper automatically labels the training dataset with vendor tag, which has better operability.

IoTTracker [28] presented a device identification method based on web page source code. The authors extracted the DOM tree as structural features and the Cascading Style Sheets (CSS) selector as style features. In the discrimination stage, the structural feature similarity is calculated based on the longest common substring between two feature sequences, and the similarity between the style feature vectors is calculated using cosine similarity. IoTTracker achieves an accuracy rate of 95.54% and a recall rate of 93.08%. This method should extract many features, which are used for device identification by calculating feature similarities. Instead, our method does not extract any features and uses CNN to obtain features automatically.

### 2.2. Unknown Class Identification

Miettinen et al. [17] proposed the IoT SENTINEL framework, which extracts 23 features from the packets when a device is first connected to the network. The RF algorithm was used to identify device types, and the identification accuracy reached 81.5%. Similarly, Sivanathan et al. [31,32] extracted features, such as flow duration, port number, DNS domain name, and cipher suite from IoT device traffic and built multi-stage classification models using NB and RF algorithms. Both methods used labeled traffic datasets, and neither considered the identification of type unseen in the labeled datasets.

Pathmaperuma et al. [40] relied exclusively on a specific threshold to determine the conversion between probability and class labels when classifying unknown applications. $P_{\max}$ is introduced to represent the highest component in the probability vector, and the sample is marked as an unknown class when $P_{\max}$ < threshold, where the value of the threshold is determined by testing on a range of candidate values. The method judges the value of the threshold by iterative testing and does not give the step size of change, so it is somehow uninterpretable.

Due to the diversity of IoT devices, it is a common scenario to meet a device whose class is unseen in the training data. Most of the literature on device identification does not discriminate device classes outside training datasets. In the few works of literature that have done this, the conclusion is made only by referring to an empiric threshold value and the accuracy is poor. This study analyzes the probability distribution of known and unknown vendors and uses machine learning algorithms to distinguish them, and the results are more meaningful and accurate.

### 2.3. Granularity of Device Identification

Feng et al. [9] proposed an Acquisitional Rule-based Engine (ARE) to automatically establish fingerprints for IoT device identification without any training data. ARE established device identification rules with the help of terms in the application-layer response data and used them as keywords to search the device description information obtained on the device website. However, when there are no keywords about device type, vendor, series, or product in the response data, the ARE method fails.

Search engines such as Shodan [6], Censys [7], and ZoomEye [8] collect many online network devices. Users can obtain fine-grained data of IoT devices through keyword retrievals, such as device vendor, type, model, location, domain name, and other information. However, the relevant technical details of these search engines have not been reported.

Due to the concise layout and clear information presentation of the device login page screenshots, it is possible to accurately extract the information about the device type, model, and other attributes on the screenshots by using OCR technology. We propose a device fine-grained device attribute identifying method based on OCR that can be used as an alternative to the above methods.

## 3. Motivation

**Figure 1:**
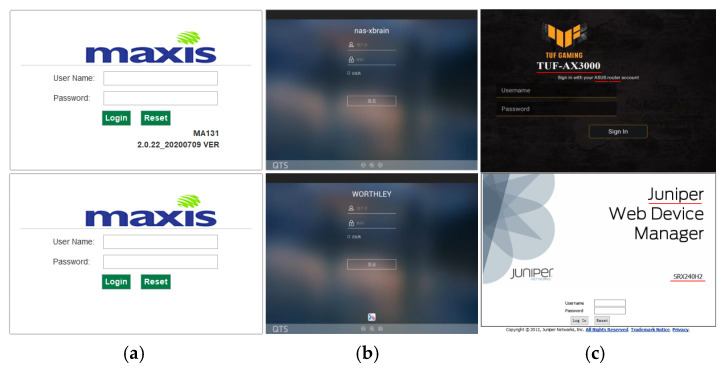
Samples of device login page screenshot: (**a**) maxis; (**b**) QNAP; (**c**) ASUS and Juniper.

Driven by standards like Web-Based Enterprise Management (WBEM), most vendors will integrate web-based management programs in their device firmware. Devices from the same vendor usually share a common style of WEB UI, while devices from different vendors usually have WEB UIs of totally different styles. As WEB UI has a high degree of recognition, it may be used as an effective feature for device vendor identification. Figure 1a shows two WEB UI login page screenshots of devices from the Maxis company, and Figure 1b shows two screenshots of devices from the QNAP company. It is easy to prove our opinion by observing these two groups of pictures, which assures us the intuition and motivation to perform device identification using image classification techniques.

Figure 1c shows two WEB UI login page screenshots that come from ASUS and Juniper company, respectively. Look out for the part on the pictures marked with underline, and you can find that both screenshots provide information about vendors (ASUS and Juniper) and models (TUF-AX3000 and SRX240H2). A screenshot from ASUS company contains explicit type information (Router), though the screenshots from Juniper company does not contain any type information, we can infer that the device is of type “firewall” by its model (SRX240H2) and corpus. Therefore, it is reasonable to extract various kinds of device attributes from the screenshots using OCR techniques.

Note that many vendors give their names in the form of some artistic logo pictures on their WEB UI login pages, which are hard to identify using OCR techniques. For example, OCR tools will identify the logo of Maxis in Figure 1b as “sixeuu” by mistake. Therefore, rather than OCR techniques, the study uses an ensemble learning model based on CNN and Res-DNN for device vendor identification.

## 4. Framework

**Figure 2:**
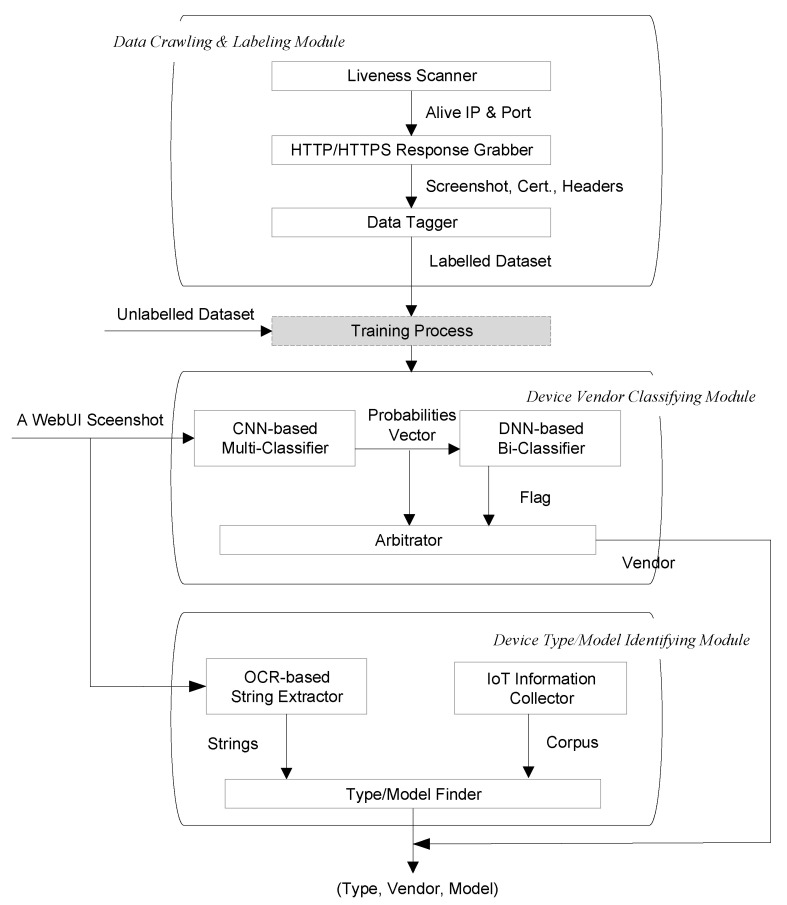
Framework for IoT device identification.

The overall framework of device identification proposed in this paper is shown in Figure 2, which can be divided into three parts: Data Crawling and Labeling Module, Device Vendor Classifying Module, and Device Type/Model Identifying Module. The Data Crawling and Labeling Module acquire device login page screenshots and automatically labels them with the device vendor to form a labeled dataset for training. The Device Vendor Classifying Module is an ensemble learning model consisting of two classifiers. The first level of the ensemble model is a CNN-based multi-classifier with screenshots as input and the output is a probability distribution over vendors of prediction for those screenshots. The second level is a DNN-based bi-classifier, which takes the probability vector generated by the multi-classifier as the input and outputs the flag to indicate whether the device belongs to the vendor in the labeled dataset. Finally, the probability vectors and flags are arbitrated to obtain the final device vendor prediction. The Device Type/Model Identifying Module extracts the strings from screenshots based on OCR technology and matches them fuzzily with the pre-established corpus of device information to obtain finer-grained attribute information such as the type and model of the device.

### 4.1. Data Crawling and Labeling

The Data Crawling and Labeling Module selects devices that support the HTTP/HTTPS protocol to build labeled image sets, which consist of Liveness Scanner, HTTP/HTTPS Response Grabber, and Data Tagger. Given the IP address segment, Liveness Scanner probes the liveness of each IP address and ports numbered as 80/8080/443/8443 commonly used for HTTP/HTTPS services. If the IP addresses and ports are alive, the HTTP/HTTPS Response Grabber uses *Selenium 3.0* and *ChromeDriver 2.38* to simulate a user accessing the device login page through a browser. A screenshot of the browser window is taken after the home page is successfully returned, and the HTTP/HTTPS response headers and/or certificates are also saved. Afterward, the Data Tagger matches the obtained response headers and/or certificates with the fingerprint entries in the *Recog* open-source fingerprint repository. If the match is successful, we will get the vendor corresponding to the device and use it to mark the device login page screenshots and save the screenshot as labeled data; otherwise, save the screenshot as unlabeled data. We will then get two datasets; one is the labeled dataset, $D_{l}=\{\left(S_{1},v_{1}\right),(S_{2},v_{2}),\cdots ,\left(S_{n},v_{n}\right)\}$, where the i-th element $\left(S_{i},v_{i}\right)$ represents the screenshot $S_{i}$ from the vendor $v_{i}$. The other is the unlabeled dataset, $D_{u}=\{s_{1},s_{2},\cdots ,s_{n}\}$, where the i-th element $s_{i}$ represents an unlabeled screenshot.

### 4.2. Device Vendor Classifying

**Figure 3:**
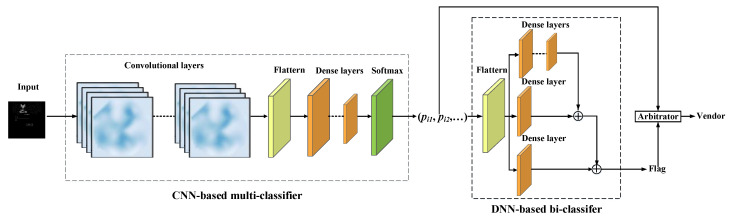
Device Vendor Classifying based on an ensemble model.

The device vendor classifying is implemented by an ensemble learning model, which consists of five components: input layer, CNN-based multi-classifier, DNN-based bi-classifier, and output, as shown in Figure 3.

The CNN-based multi-classifier takes screenshots from the dataset $D_{l}$ as input, and the input layer is connected to convolution layers to learn the features of the screenshots. We use three convolution layers, each of which is followed by batch normalization, an activation function, and a pooling layer to select the most prominent features and reduce the number of parameters. The max-pooling function is chosen for the pooling layer. The outputs of the last convolutional layer are flattened and passed to dense layers. We designed five dense layers, and the first four dense layers are followed by the activation function Rectifier Linear Units (ReLU. The final activation function is SoftMax, which outputs a multi-dimensional probability vector. The probability vector is a numerical vector whose entries range from 0 to 1. The number of classes determines the number of entries in the multi-dimensional vector, and the final classification result depends on whether the class corresponds to the maximum value in the entries.

The multi-classifier can identify the classes based on the dataset $D_{l}$. Suppose there is a real-world screenshot from the dataset $D_{u}$ that has not been trained, which obviously cannot be correctly classified by the multi-classifier. To be able to accurately discern whether a screenshot is from $D_{l}$ or $D_{u}$, we label the data in $D_{l}$ as known, label the data in $D_{u}$ as unknown and merge the two into a new dataset. The new dataset is fed into the multi-classifier to obtain the result set, $R=\{(P_{1},k_{1}),(P_{2},k_{2}),...(P_{n},k_{n})\}$ where $\left(P_{i},k_{i}\right)$ is a tuple consisting of the probability vector $P_{i}=<p_{i1},p_{i2},\cdots >$ predicted by the multi-classifier for the i-th screenshot and the corresponding label $k_{i}$ (either known or unknown) of the i-th screenshot. We train a bi-classifier with *R* as the input to discriminate whether a screenshot belongs to a known or unknown class. For each screenshot, CNN output a probability vector of 30 dimensions, and each probability vector belongs to a hyperplane according to the class of its related screenshot. The neurons in the Res-DNN neural network can fit the hyperplane so as to update the parameters of Res-DNN, making Res-DNN capable of classifying the probability vector output by CNN.

In Res-DNN, we connect three network branches composed of different numbers of fully connected layers using skip connect. The first branch has four fully connected layers, and the second and third branches have only one fully connected layer, respectively. Each fully connected layer consists of sense layers, Batch Normalization1D, and activation function. The predictive output of the fully connected layer is expressed as $pred=<y_{{}_{0}},y_{1},\cdots ,y_{n}>$, where the output of the j-th neuron is expressed as Equation (1), and $p_{i}$ is the i-th component of a probability vector P, and n is the dimension of vector input to the current fully connected layer.

(1) $$y_{j}=\mathrm{ReLU}(\sum_{i=1}^{n}\left(\omega_{ij}p_{i}+b_{ij}\right))$$

The predictive results of three DNN branches ($pred_{1}$, $pred_{2}$ and $pred_{3}$) are connected by skip connect according to Equation (2). We use the binary cross-entropy function shown in Equation (3) as the loss function of Res-DNN, where N is the number of samples in the current training batch.

(2) $$pred=pred_{1}+pred_{2}+pred_{3}$$

(3) $$\;loss=\frac{1}{N}\sum -[k_{i}\cdot \log(pred)+(1-k_{i})\cdot \log(1-pred)]$$

The bi-classifier is a skip connect that connects three network branches composed of fully connected layers together, and we call this connection method Res-DNN. The first branch has four fully connected layers, whereas the second and third branches have only one. Each fully connected layer consists of a dense layer, a batch normalization, and an activation function. The bi-classifier takes the probability vector R generated by the multi-classifier as the input, and the output is a binary flag to indicate whether the screenshot belongs to a known class. If the flag is true, the vendor corresponding to the maximum component of the probability vector output by the multi-classifier is deemed as the final result; else, the vendor of the screenshot is considered unknown.

### 4.3. Device Type/Model Identifying

**Figure 4:**
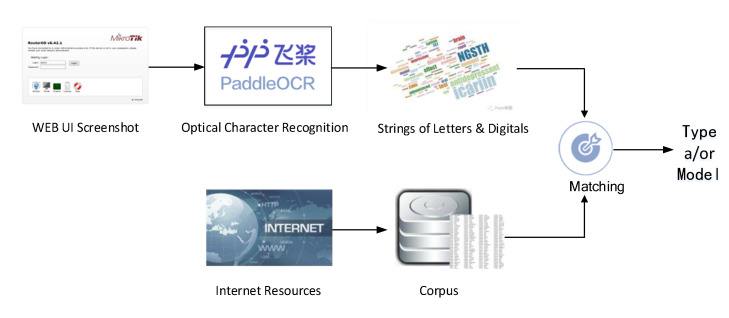
Device Type/Model Identifying based on OCR.

Since most IoT device screenshots contain information such as the type and/or model, this paper proposes an OCR-based method for device fine-grained attribute identification. The main workflow is shown in Figure 4.

Firstly, an IoT device corpus is constructed, which contains the device vendor, type, and model. The device type is obtained from the list of mainstream device types provided in the open-source fingerprint repository *Recog*. Considering the variety of device models and the high cost of manual collection, we use a crawler program to automatically extract and collect device models from the device-related web pages of e-commerce platforms.

Secondly, we use Paddle OCR [46], a lightweight image-to-text toolkit integrating many detection and recognition algorithms, for textual information extracted from screenshots. To improve the efficiency of matching between the extracted text and the entries in the corpus, text strings containing characterless words (login, username, password, etc.) are removed.

Finally, the entries in the corpus are matched with the strings extracted by OCR based on the longest common subsequence (LCS) algorithm. Considering the inaccurate recognition that Paddle OCR may have when the screenshot resolution is poor, we introduce ratio defined by Equation (4) as an indicator to measure whether the matching is acceptable, where $L_{corpus}$ is the length of an entry in the corpus $L_{ocr}$ is the length of a string extracted by OCR, and $L_{lcs}$ is the length of the LCS of the entry and the string.

(4) $$ratio=(\min(L_{corpus},L_{ocr})-L_{lcs})/\min(L_{corpus},L_{ocr})$$

The range of ratio is between 0 and 1, and ratio=0 means a perfect match and ratio=1 means a total mismatch. In practice, we choose a threshold of 0.1 and accept a match between entries in the corpus and strings extracted by OCR when ratio≤0.1.

## 5. Experiments and Evaluation

### 5.1. Data Preparation and Parameter Setting

We grabbed device login pages from several IP address segments of the B class and tried tagging them with vendor information obtained by fingerprint matching or from SSL certificates, and finally got a dataset $D_{l}$ of more than 36,000 pieces of screenshots labeled with vendors. The screenshots in the dataset $D_{l}$ come from 30 vendors and a broad range of device types, including camera, router, firewall, etc., as shown in Table 1. The *Recog* open-source fingerprint repository only contains about 5000+ fingerprint entries, so there exist some devices that cannot be recognized and labeled due to the capability of *Recog*. We totally get 7000 such devices that cannot be labeled and put into set $D_{u}$ representing devices from the unknown world. To overcome the data imbalance problem, we perform (Synthetic Minority Oversampling Technique) SMOTE [47] on samples from the same vendor but less than 1000.

The resolution argument in *ChromeDriver* is set to 1024 × 768, which is a common 4:3 ratio and is suitable for screenshot capture of all the WebUI of devices in the experiment. Screenshots too large may reduce the speed and accuracy of image analysis and processing [39] and may also lead to a decrease in the training efficiency of the entire neural network, so we transform the screenshots into images of 224 × 224 × 3 RGB with data enhancement.

The input image size of the CNN is 224 × 224 × 3, where 224 × 224 is the dimension of the image following empirical tests. We set the size of the convolutional kernels in the convolutional block to be monotonically non-increasing and the number to be monotonically non-decreasing. Therefore, the shallow neural block has a smaller number of channels and a larger convolutional kernel size, and the deeper convolutional block has a larger number of channels and a smaller convolutional kernel size to extract data features from different network levels. The detailed parameters of the CNN and Res-DNN are shown in Table 2.

### 5.2. Effects of Vendor Identification

The CNN model is rained from the dataset $D_{l}$ using five-fold cross-validation. Then, $D_{u}$ and the test set in $D_{l}$ are input to CNN, whose output is used as a new data set to train the Res-DNN model to distinguish between known and unknown vendors. The dataset $D_{l}$ is divided equally into five parts for the five iterations of training and prediction to be performed. In each iteration, three parts of data are used for training, one part for validation, and one part for the test. After iterations, five models are trained and five sets of predictions are obtained. Then, the dataset $D_{u}$ is fed as test data to each model trained to obtain five sets of predictions. Finally, all the above predictions as new datasets are split into 80% training and 20% testing datasets to train the Res-DNN model.

#### 5.2.1. Multi-Classifier

We analyzed the performance of CNN-based multi-classifiers using Accuracy, Precision, Recall, and F1-Score metrics. F1-Score is the weighted average of Precision and Recall, which takes both false positives and negatives into account, and is more indicative of the classification performance, especially when the data are unevenly distributed. Table 3 performs a five-fold cross-validation. The accuracy and precision of the models for every cross-validation reached more than 97.8%, and the recall and F1-Score reached were greater than 99%. After calculation, this multi-classifier has an average accuracy of 0.98% and an averaged F1-Score of 0.983%.

**Figure 5:**
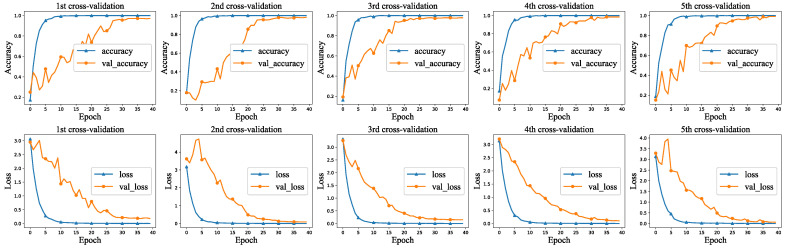
Multi-classifier training and validation visualization.

The multi-classifier model has high average accuracy and well maintains stability during the training. Figure 5 shows a visual representation of the training and validation. The identification accuracy of the devices keeps improving gradually, and the loss value decreases steadily and reaches a stable state after the 30th epoch of training.

**Figure 6:**
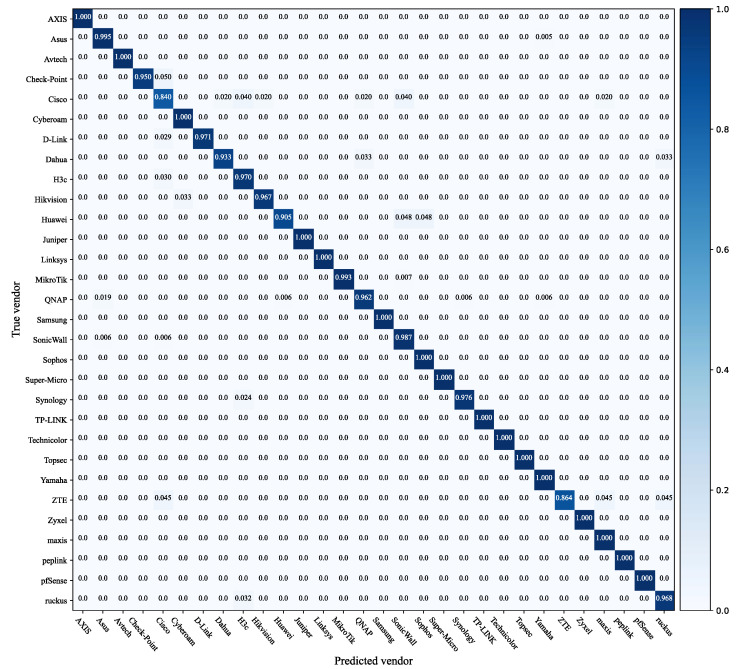
Multi-classifier confusion matrix.

The confusion matrix of the classifier is shown in Figure 6, which describes the multi-classifier in detail. Classification accuracy can be defined as the ratio of the correct device vendors to the total number of predictions. In the confusion matrix, the elements on the main diagonal represent the probability of correct classification of the test sample, while the elements on the off-diagonal represent the probability of incorrect classification. Fifteen vendors, such as Avtech, Cyberoam, Juniper, etc., could be classified completely correctly. A few Cisco devices were misclassified as Dahua, H3c, Hikvision, etc. A few ZTEs were misclassified as Cisco, Maxis, and Ruckus. The multi-classifier we built can effectively discriminate the device vendors for the labeled screenshots.

#### 5.2.2. Bi-Classifier

**Figure 7:**
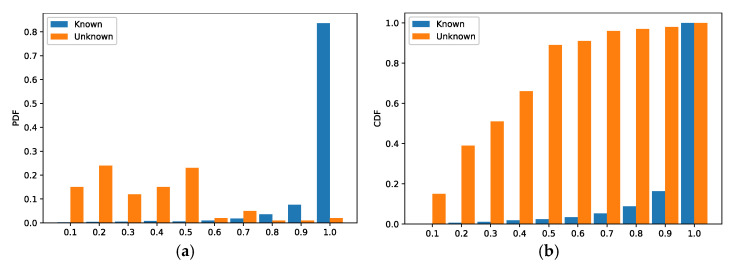
Evaluation results of the multi-classification model. (**a**) PDF (**b**) CDF.

We use a CNN-based multi-classifier to classify samples from the dataset $D_{l}$ whose vendor depends on the class of the maximum value of the probability vector output by the CNN. The classes of samples from the dataset $D_{u}$ cannot be correctly predicted by the trained classifier. To observe the probability distribution of the dataset $D_{l}$ and $D_{u}$, we input them to the CNN model to obtain two sets of probability vectors. Next, we analyzed the two sets of probability vectors using both the Probability Density Function (PDF) and the Cumulative Distribution Function (CDF). As shown in Figure 7, the samples from $D_{l}$ are labeled as known and the samples from $D_{u}$ as unknown. In Figure 7a, the X-axis represents the dispersion interval of the maximum value of the probability vector output from the CNN, and the y-axis represents the probability that $P_{\max}$ falls into a certain interval. In Figure 7b, the x-axis has the same meaning as that of Figure 7a, and the Y-axis represents the accumulation of the probability that $P_{\max}$ falls into a certain interval. As shown in Figure 7a, for samples of known class, the $P_{\max}$ of 85% of the samples are concentrated in the interval [0.9, 1], while for samples of unknown class the $P_{\max}$ of most samples are scattered in the interval [0.1, 0.5]. It is obvious that there is a significant difference in the distribution of $P_{\max}$ between the samples from the dataset $D_{l}$ and the samples from the dataset $D_{u}$, so it is reasonable to choose a probability vector as input for the training of the binary classifier.

**Figure 8:**
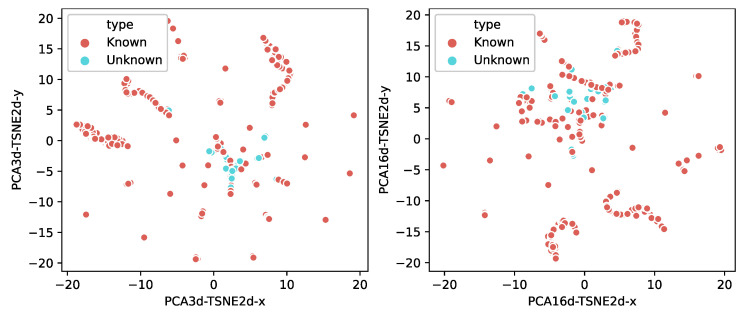
Visualization of the probability vectors from the multi-classifier.

To demonstrate that the known and unknown samples are distinguishable, we visualized the probability vectors of the test samples. Since the probability vectors are multidimensional (i.e., each data includes 30 entries) and not easy to visualize directly, we used the Principal Component Analysis (PCA) [48] and the t-Distributed Stochastic Neighbor Embedding (t-SNE) [49] dimensionality reduction techniques to project the data onto two dimensions. As shown in Figure 8, the left figure is first reduced to three dimensions by PCA and then to two dimensions using t-SNE. The right figure is first reduced to 16 dimensions by PCA and then to two dimensions using TSNE. From the two figures, we find that the distribution of the maximum values from known samples is discrete, while the distribution of the unknown samples is very concentrated. The distributions indicate that the known and the unknown samples are separable.

We learn the distributions of known samples and unknown samples on the proposed Res-DNN model. The performance of the Res-DNN-based bi-classifier reaches an average accuracy of 99.1% and an F1-Score of 99.5%. It illustrates that the Res-DNN bi-classifier can distinguish known and unknown vendors, and the vendors of the samples judged as known are subject to the results predicted by the multi-classifier. We also test 7 other machine learning algorithms such as Logistic Regression (LR), Decision Tree, KNN, SVM, TCN, RF, and Wide Residual Networks (WideResNet) on the same dataset. The accuracy, precision, recall, and F1 score of each algorithm are shown in Table 4. The results show that when distinguishing known vendors from unknown vendors, all the performance indexes of these algorithms reach more than 94%. Among them, the Decision Tree algorithm performed the worst, with its F1-Score of only 94.2%. These experimental results show that our proposed Res-DNN can not only distinguish known and unknown vendors but also outperforms other models.

### 5.3. Effects of Fine-Grained Identification

To test the precision of fine-grained identification of IoT devices, we used the API provided by the Shodan search engine to obtain 8000 IP nodes and their corresponding device information using the vendor name “ASUS” as the keyword. We used these IPs to crawl the login page screenshots on the Internet and obtained 7017 valid screenshots. Next, we used the PaddleOCR SDK to extract text from the 7017 screenshots and matched them with the corpus. Note that although vendor identification is not the main purpose of this fine-grained identification experiment, we still identify the vendor in the screenshot.

**Figure 9:**
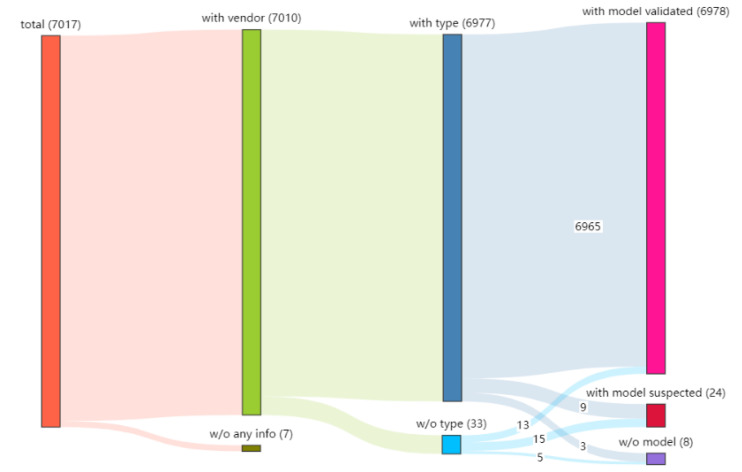
OCR-based identification results.

As shown in the Sankey diagram in Figure 9, we found seven screenshots that do not contain any device information, and the reasons may be that there is no textual information in the screenshots or the extracted text could not be matched with the corpus. The remaining 7010 screenshots contained vendor information, of which 6977 contained both vendor and type information and 33 had no type information. Six thousand nine hundred seventy-eight screenshots contained model information and were successfully matched with the corpus; 24 screenshots contained suspected model information (strings composed of letters and digits) but were unsuccessfully matched with the corpus, probably due to incomplete information in the corpus. Eight screenshots did not contain any model text (e.g., only vendor or type information). We can confidently identify the vendor, type, and model from the screenshots. In the case of model information only, the vendor and type information can also be obtained by inference. We can confidently identify the manufacturer, type, and model of the device from the screenshots on the login page. In the case of model information only, the manufacturer and type information of the device can also be obtained by inference. Finally, we counted 20 vendors, 10 types, and 112 models from 7017 screenshots.

We compare our identification results with that of Shodan and find that, of these 7017 devices, Shodan has got 606 errors in vendor identification, 187 errors in type identification, and 231 errors in model identification. For example, Shodan may identify by mistake Ruijie, Netgear, and H3C as ASUS devices, identify ASUS RT-AC5300 as ASUS RT-AC68U, and PHICOMM-K3 as RT-AC66U B1, etc. The accuracy of the OCR-based method and Shodan was compared, as shown in Table 5. If the method proposed by us is adopted, the accuracy of identification of vendors, types, and models can be improved by 8.54%, 2.28%, and 11.37%, respectively.

Experiments show that the device type and model information is contained in the device login pages. In the case of rich corpus information, the device fine-grained identification method can accurately extract the device model information and find the match in the corpus, then identify the device in finer granularity.

## 6. Conclusions

In this paper, we propose a novel and alternative method for IoT device identification, which introduces commonly available login pages with distinctive characteristics specific to vendors as the data source and uses an ensemble learning model based on a combination of CNN and Res-DNN for device identification and develops an OCR-based method for device type and model identification. The experimental results show that the ensemble learning model can achieve 99.1% accuracy and 99.5% F1-Score in the determination of whether a device is from a vendor that appeared in the training dataset, and if the answer is positive, 98% accuracy and 98.3% F1-Score in identifying which vendor it is from. The OCR-based method can identify fine-grained attributes of the device and achieves an accuracy of 99.46% in device model identification, which is higher than the results of the Shodan cyber search engine by a considerable margin of 11.39%. We will collect WebUI screenshot samples from more manufacturers, establish a more richer device corpus, and implement an open-source and usable system prototype based on existing methods to make more practical contributions to the field of IoT identification. The method proposed in this paper can support the classification and identification of IoT devices from 30 manufacturers. However, due to the large number of IoT equipment manufacturers and the continuous emergence of new manufacturers, how to propose an open and rapidly scalable classification model will be an important research direction for us in the future.

## Figures and Tables

**Table 1:** 30 IoT vendor devices.

Vendor	Type	Quantity	Vendor	Type	Quantity
AXIS	Camera, etc.	1769	Samsung	Camera	1597
Asus	Router	1431	SonicWall	Firewall	1286
Avtech	Camera	1301	Sophos	Firewall	2541
Check-Point	Firewall, etc.	1523	Super-Micro	Gateway	1862
Cisco	Router, etc.	1409	Synology	NAS	1131
Cyberoam	VPN	354	TP-LINK	Router, etc.	1747
D-Link	Router, etc.	678	Technicolor	Gateway	1032
Dahua	DVR, etc.	635	Topsec	Firewall	1428
H3c	Firewall	685	Yamaha	Network switch	527
Hikvision	Camera, etc.	871	ZTE	Router, etc.	2086
Huawei	Switch, etc.	2378	Zyxel	Router, etc.	740
Juniper	Firewall	1023	maxis	Router	462
Linksys	Router, etc.	856	peplink	Router	791
MikroTik	Router	385	pfSense	Firewall	692
QNAP	NAS	2863	ruckus	Access Controller	563

**Table 2:** Parameters configuration of the CNN and Res-DNN.

Classifier Model	CNN	Res-DNN
Input size	224 × 224 × 3	16 × 16
Convolutional kernel number	16, 32, 64	-
Convolutional kernel size	7 × 7, 3 × 3, 3 × 3	-
Pooling type	Max pooling	-
pool_size	(22)	-
Optimizer	SGD	Adam
Sizes of dense layers	1000, 256, 128, 64, 30	L1: 1000, 500, 150, 10, 2; L2: 30, 2; L3: 60,2
Bach_size	128	128
Momentum	0.9	-
Learning Rate	0.005	0.002
Activation function	ReLU, SoftMax	ReLU
Loss function	Cross entropy	Cross entropy

**Table 3:** Performance of multi-classifiers in IoT device identification on cross-validation.

Times	Accuracy	Precision	Recall	F1-Score
1st cross-validation	0.978	0.987	0.980	0.983
2nd cross-validation	0.984	0.986	0.990	0.988
3rd cross-validation	0.978	0.984	0.983	0.983
4th cross-validation	0.981	0.980	0.987	0.983
5th cross-validation	0.978	0.977	0.980	0.978
average	0.98	0.983	0.984	0.983

**Table 4:** Performance of the classifiers in distinguishing between the known and unknown vendors.

Model	Accuracy	Precision	Recall	F1-Score
Logistic Regression	0.957	0.956	1	0.9786
Decision Tree	0.969	0.990	0.8977	0.984
KNN	0.976	0.992	0.983	0.987
SVM	0.980	0.996	0.983	0.989
TCN	0.985	0.996	0.989	0.992
RF	0.987	0.991	0.994	0.993
WideResNet	0.989	0.990	0.996	0.994
Res-DNN	0.991	0.992	0.998	0.995

**Table 5:** Comparison of OCR-based method and Shodan.

Attributes	# by Our Method	Accuracy of Our Method	# by Shodan	Accuracy of Shodan
Vendor	7010	99.9%	6411	91.36%
Type	6990	99.62%	6830	97.34%
Model	6978	99.46%	6786	88.07%

## Data Availability

The data used to generate the plots and figures can be accessed by contacting the author at dongxinbaoer@163.com.
